# Assessing client needs in community veterinary care: a case study from WisCARES

**DOI:** 10.3389/fvets.2025.1675984

**Published:** 2025-10-13

**Authors:** Kelly Schultz, Elizabeth Alvarez, Jennifer Brooks, Ruthanne Chun

**Affiliations:** School of Veterinary Medicine, University of Wisconsin, Madison, WI, United States

**Keywords:** accessible veterinary care, pet food, veterinary dental care, affordable grooming services, veterinary social work, community veterinary care, social services, access to care

## Abstract

As community veterinary clinics expand across the United States, there is a growing recognition that services need to be aligned with the specific needs of clients and their companion animals, which may vary from one community to the next. WisCARES Community Clinic, which has served low-income pet owners in Dane County, Wisconsin for over a decade, conducted a comprehensive needs assessment in the summer of 2024 to re-evaluate the support required by its clientele. Through an interviewer-administered survey of 51 clients, conducted either in person or by phone, the study identified key areas for assistance. Clients most frequently reported needing support with pet food, treats, pet cleaning supplies, and dental care items, as well as access to affordable grooming services. In addition, many clients expressed a need for help navigating social services and securing reliable transportation. These findings highlight that clients experiencing poverty require more than basic veterinary care to maintain the health and well-being of their pets. While local contexts vary, this assessment offers valuable insight for other community veterinary programs seeking to allocate limited resources to where they will have the greatest impact.

## Introduction

Nearly half of United States households own at least one dog and a third own at least one cat (1). According to data from the Humane Society of the United States (2), 43% these households reported that they were unable to afford their pets’ care at some point, revealing a need for accessible veterinary care. While some people struggle to understand why others have pets if they cannot afford to care for them, a wealth of research shows that many people are healthier when they share their lives with animals (3–6). In addition to these benefits, the realities of life for people in lower income brackets mean that they are also more likely to seek dogs for protection (7). People in lower socioeconomic status (SES) brackets are more likely to both get and to give companion animals to friends and family (8) when times are tough. Obtaining a new pet is not always planned but having one may strengthen the social capital amongst family and community (9). Regardless of how animals come into families, community medicine and accessible veterinary care programs provide alternatives to surrender, early euthanasia and unnecessary suffering due to economic factors (10).

The term ‘One Health’ indicates that the health of animals, humans and the environment in which they live is intertwined (11). Broadly, pet companionship is associated with increased physical activity along with better mental and emotional health (12, 13). Access to companion animal care may lead to healthier and more connected community members and is an example of a One Health approach to care. However, living closely with pets can also carry risks, such as conditions ranging from internal and external parasites to bacterial, fungal or viral infections may be passed from companion animals to people (14, 15). Helping a family keep a pet (and subsequently keeping that pet healthy) can be viewed as an extension of One Health community medicine.

Community medicine originated from the public health and social medicine movement that began in the mid-nineteenth century when people migrated en masse to cities during the industrial revolution (16). The modern approach is characterized by the practice of using collaborative medical approaches to increase both the promotion of healthy behaviors and the prevention of disease on an individual level (17). Community medicine focuses on individual patients and emerges from a holistic understanding of the factors underlying community health outcomes for a defined community. These outcomes arise from evidence-based approaches that integrate medicine, public health science, cultural humility, and goal setting around optimization of health and quality of life (18).

Veterinary health care adopted these core definitions and in the 2010s began integrating community medicine and accessible veterinary care into the practice of companion animal medicine (19). The underlying goal is to create systems in which veterinary care is financially and logistically within reach and is tailored to the community in which the clinic is situated. While barriers to accessibility are often financial, there are additional factors (20) that determine whether a companion animal guardian is able to access veterinary care. These include having the ability to be compliant with medications, the time to attend recheck appointments, the availability of transportation, and the ability to understand and retain the details of disease management (21, 22).

Local biannual Point in Time surveys document 600–800 unhoused people sleeping in shelters or encampments in Madison, Wisconsin. The United Way’s Asset Limited Income Constrained Employed (ALICE) report states that 23% of Dane County families (roughly 57,519 households) are living paycheck to paycheck; this population is employed and earns above the federal poverty level but does not make enough money to afford a basic household budget (23). Knowing that around half of these families care for at least one dog or cat, the WisCARES outreach program was developed to address the community’s need for free or low-cost quality veterinary care.

WisCARES (Wisconsin Companion Animal Resources, Education, and Social Services) at the University of Wisconsin (UW)—Madison, was established in 2013 as a collaboration among the Schools of Veterinary Medicine, Pharmacy, and Social Work. WisCARES is a unique interdisciplinary program that aims to: increase access to veterinary services for low-income and homeless pet owners; provide concurrent social services and human health care support to the same clientele; and engage participating students with practical interprofessional service learning in a One Health setting (24).

WisCARES is a full-service veterinary clinic, open 5 days a week, situated in a lower socioeconomic (low-SES) zip code area. The clinic provides preventative, routine, and urgent veterinary medical care, as well as dentistry and surgical procedures, for pets whose owners qualify (24). Nearly all of our clients fall below 200% of the federal poverty line, with a few exceptions for clients who may be navigating domestic abuse or who qualify for aid based on other factors. Roughly 40%–50% of our clients report that they are people of color, 10%–20% report being LGBTQA+ and 60%–70% report having a disability (physical, cognitive, learning or emotional). Student participation exists primarily as formal internship opportunities for veterinary medical and social work students. Fourth-year veterinary students enroll in 2-week clinical rotations, while undergraduate and graduate social work students enroll in field placements for a full academic year. All students receive academic credit for their internships. Pharmacy and nursing students volunteer more informally, through a student-run club, rather than completing an academic internship. The final type of student participation at WisCARES is part-time, paid employment as a veterinary assistant. UW pre-veterinary and social work students have been employed in this capacity. The result is an interprofessional clinic that provides compassionate care for the entire family unit, offering greater patient/client support and bridging potential gaps in care (25).

In addition to providing subsidized veterinary medical care to animals owned by Dane County families experiencing homelessness and/or poverty, WisCARES offers a petfood pantry, pet supplies, housing advocacy (both to prevent eviction and to find housing) as well as animal fostering. There is a wealth of social services in Dane County, and yet people in need are not always able to find them. In these situations, the social work team works with interested clients in a variety of situations, such as helping with job interviews, medical appointments, and meeting with landlords.

In a typical year, WisCARES sees about 3,000 cases, works with between 70 and 100 students, gives roughly 2,500 vaccines and moves about 10 tons of affordable to free pet food into the community. Because WisCARES is a brick-and-mortar veterinary facility that also acts as a hub for dispersing donated supplies, while helping people find medical resources and helping people with social services, the staff at the clinic also field between 50 and 100 phone calls and emails each day. Of the clients that come to WisCARES, roughly 70% engage with the social work team at one time or another.

WisCARES expands service provision in an intentional, sustainable fashion in response to clients’ needs (for veterinary care, routine pet care and social assistance), while also ensuring that veterinary services available elsewhere in the community are not duplicated. For example, in the instance of the Dane County community, there are multiple locations for low cost spays and neuters through both for-profit and not-for-profit organizations. As a result, WisCARES offers this service in a limited capacity, but does not elevate it to a predominate goal of the clinic. This is important as a protection of the limited resources of the clinic, but it is also important as a responsible member of a community of businesses. In other locations, spays and neuters may be needed as a predominate feature of community support. As a community-based organization, it is important to learn about these needs directly from clients rather than claiming the expertise to make decisions for them (26). Clinic staff track the services clients request while in the clinic, conduct client satisfaction surveys, monitor online reviews, and perform formal needs assessments. Needed and underutilized services are identified through these varied channels. The goal is to effectively steward limited resources (27).

In 2024, WisCARES conducted a client needs assessment hoping to identify ways to expand both clinic services and our Community Pet Resource Center’s offerings. This survey-based assessment included questions about current and potential services, and participants were invited to suggest additional services for themselves or their companion animals through open-ended questions.

## Methods

### Survey participants

Surveys were performed from May to October 2024. In-person interviews were conducted with clients while they were waiting to pick up their pets at the WisCARES clinic. Telephone interviews were conducted from randomly generated lists of clients who had utilized any WisCARES services within the past 12 months and clients who had not visited WisCARES within the past 12 months. Participation was voluntary; consent was indicated by verbally electing to continue with the survey. The University of Wisconsin-Madison Education and Social-Behavioral Science Institutional Review Board deemed this study to be exempt from IRB oversight.

### Survey design

Survey questions were designed to evaluate the needs of cat and dog owners. Respondents were provided opportunities or prompted to elaborate on items described as useful to them. Prompted responses were divided into the following categories: supplies for cats (food, litter, toys, etc.), supplies for dogs (food, leashes, toys, etc.), animal service needs (training classes, pet grooming, foster care, etc.) and human services/supplies (finding local agencies, food support, legal resources, etc.). Respondents who identified as cat owners, were delivered the prompts for cat supplies, while dog owners were delivered the prompts for dog supplies. Respondents reporting owning both dogs and cats received both portions. Interviewers asked a mix of open-ended questions and closed-ended prompts (Supplementary Appendix S1). Open-ended questions were asked first to hear respondents’ unbiased, top-of-mind thoughts. Prompted questions followed, with lists of items and services that are currently available, as well as those that are not available but have been requested by clients or suggested by staff. At the end of the survey respondents were again asked for open-ended responses about items and services in case their responses changed after hearing the options listed in the prompted questions. To gather feedback on survey content, terminology, and understanding, with the intent of achieving face validity (i.e., the degree to which the survey appeared effective in its objective), the survey was pre-reviewed by a convenience sample of three academic access-to-care veterinarians, a veterinary nurse, and two social workers. Members of the University of Wisconsin-Madison Survey Center then reviewed all survey questions for clarity. The survey was generated in Qualtrics. Questions were read in person or via phone interviews to survey participants by paid WisCARES personnel. Verbal responses were entered directly into Qualtrics, so data was captured electronically (Qualtrics software, Version XM).

### Statistical analysis

One investigator undertook initial theme coding, then compared the results. Coding was undertaken by reading through the written responses and noting initial themes. All open-ended questions were tabulated as lists of needs by the survey administrators to avoid documenting stream of consciousness responses. These lists of responses were then categorized and placed into groups of similar answers. Answers to open-ended questions were categorized and placed into themes, when applicable. All basic descriptive statistical analyses were performed with Excel (version 16.77) and R (version 4.3.1).

## Results

### Demographics

A total of 51 surveys were completed. Eighteen in-person surveys were conducted while owners were waiting in the clinic; the total number of clients asked to take the survey in person was not recorded. Surveyors called 100 clients who had utilized WisCARES in the previous 12 months and of these 27 (27.0%) people completed the survey. Surveyors called 45 clients who had not utilized WisCARES for at least 12 months and of these 6 (13.3%) people completed the survey. Of people who had not been to the clinic in over a year, 31% (14/45) had phone numbers that no longer worked. In contrast to this, only 8% (8/100) of people who had been to the clinic within the last year had phone numbers that no longer worked.

Of the 51 respondents, 40 (78.4%) owned at least one dog and reported having an average of 1.4 dogs per family. Out of our total respondents, 25 (49.0%) owned at least one cat and reported having an average of 2.0 cats per family. Fourteen (27.5%) respondents owned both dogs and cats and two individuals reported owning another type of small mammal (e.g., rat or hamster) in addition to having at least one dog and cat. Families surveyed owned 2.1 companion animals on average.

### Records consistency

Owners were asked how many pets they had at home and, before anonymizing the data, the number of animals that were in the WIsCARES electronic medical record (EMR) was recorded by the surveyor. For 36 (70.6%) of the respondents, the number of animals reported at home matched the number of animals within the EMR. Of the remaining 15 respondents, 11 (21.6%) had one more or one less pet than the number of animals with medical records. Of the remaining 4 (7.8%) respondents, there were between 2 and 6 pets reported that were not registered in the EMR.

### Obtaining supplies

**Figure 1:**
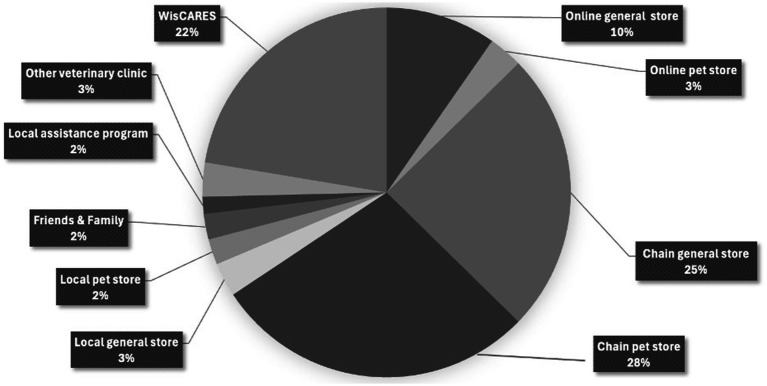
Pie chart depicting the 10 predominate sources from which WisCARES clients obtain pet supplies. Percentages were obtained from 134 responses given by 51 clients.

Clients were given the prompt: *“People get the things they need for their pets from all kinds of places. In the past year, what kinds of stores, clinics, agencies, family, or friends have you gone to, to get the things you need for your pet?*” People were able to provide as many answers as they wanted and gave a total of 134 answers (range: 1–5 answers). Answers were then grouped into ten categories with the following frequencies of response: chain pet store (28%), chain general store (25%), WisCARES (22%), online general store (10%), online pet store (3%), local general store (3%), other local veterinary clinics (3%), local pet store (2%), local assistance programs (2%) and friends and family (2%) (Figure 1).

### Close-ended prompts

#### Cat and/or dog supplies

Clients were asked to identify which supplies they would utilize if they were provided by the WisCARES clinic (Table 1). Cat owners (*n* = 25) were asked, *“Which of these ‘CAT’ supplies would you personally use if they were available at WisCARES?”* Next, participants were asked to select up to12 categories of cat supplies. Clients provided 193 affirmative responses to these prompts. The top 5 items selected were cat treats (76.0%), cat food (76.0%), scratching surfaces (72.0%), preventative dental supplies (72.0%), and cat litter/litter boxes (72.0%). Dog owners (*n* = 40) were asked, *“Which of these ‘DOG’ supplies would you personally use if they were available at WisCARES?”* Next, they were read 14 categories of dog supplies. Clients provided 353 affirmative responses. The top six items selected were dog food (77.5%), dog treats (75.0%), shampoos and cleaners (72.5%), preventative dental supplies (67.5%), outdoor apparel (67.5%), and collars/harnesses (67.5%).

**Table 1:** Table showing response rates to prompted categories of needs.

	Number	Percent
Cat supplies (*n* = 25)		
Cat food pantry	19	76.0
Treats	19	76.0
Cat litter/cat boxes	18	72.0
Preventative dental supplies	18	72.0
Scratching pad/board/posts	18	72.0
Cat trees	16	64.0
Bed	15	60.0
Carrier/crates	15	60.0
Toys	15	60.0
Food/water bowls	14	56.0
Nail trimmers	14	56.0
Collar/harnesses	12	48.0
Dog supplies (*n* = 40)		
Dog food pantry	31	77.5
Treats	30	75.0
Basic shampoo or cleaners	29	72.5
Collar/harness	27	67.5
Outdoor apparel	27	67.5
Preventative dental supplies	27	67.5
Bed	26	65.0
Food/water bowls	26	65.0
Toys	26	65.0
Nail trimmers	25	62.5
Hair clippers	23	57.5
Leash	23	57.5
Carrier/crate	17	42.5
Pet diapers	16	40.0
Animal service (*n* = 51)		
Pet grooming	40	78.4
Consultation with an animal behaviorist	28	54.9
Dog training classes	28	54.9
Boarding	24	47.1
Doggy day care services	23	45.1
Temporary foster care	15	29.4
Human service (*n* = 51)		
Find out about local agencies and social services	27	52.9
Transportation support	23	45.1
Food support	21	41.2
Join a WisCARES pet owner forum	20	39.2
Warm weather supplies	20	39.2
Cold weather supplies	19	37.3
Help talking with landlords	19	37.3
Legal resources	19	37.3
Personal hygiene supplies	16	31.4
Navigate medical or mental health appointments	13	25.5
Help with navigating job interviews	10	19.6
Finding child daycare support	9	17.6

Responses are given in total number of affirmative responses and percent of total clients who replied with an affirmative response.

#### Animal services

All clients (*n* = 51) were asked, “Which of these ‘animal services’ would you personally use if they were available at WisCARES?” Six different categories of animal services were listed. Clients provided 158 affirmative responses. The top three categories of animal services clients identified wanting support included pet grooming (78.4%), dog training (54.9%), and behavior consultations (54.9%). Owners were asked to elaborate on what aspects of these services they needed. Of the 40 owners who selected pet grooming as an available resource for their pet, five (12.5%) needed nail trims and three (7.5%) needed bathing. Additional respondents reported examples such as removing hair mats, cleaning ears, emptying anal sacs, or wanting to learn grooming techniques. Owners who stated that they wanted other services (dog training, behavior consults and day care) often cited that they wanted these services to help socialize their pet and reduce their pet’s anxiety/aggression towards other dogs or people. Clients also stated that they wanted obedience training sessions to help with pet recall, basic commands and housebreaking.

#### Human services

All clients (*n* = 51) were asked, “*Which of these ‘human services’ and supplies would you personally use if they were available at WisCARES?*” Twelve categories of human services were listed. Clients provided 216 affirmative responses. The three most common requests were, “help finding information about local agencies and social services” (54.9%), “transportation support” (45.1%) and “food support” (41.2%). Owners were asked to elaborate on what aspects of these services they needed. Nineteen clients (37.3%) said they needed help with landlords and of these, seven (36.8%) clients said they specifically needed help finding housing that would allow their particular pets. Further clarifying this request, respondents cited worries of breed specific housing rules, general disallowance of pets and worries about their dog’s excessive barking. Clients who asked for help obtaining food for themselves or their families reported that they needed help accessing food stamps and food pantries. Clients who requested transportation assistance discussed needing help with using the bus with their pet, paying for gas and difficulty transporting large dogs.

#### Open-ended responses

Again, to ascertain unbiased responses, participants were asked to name desired services and supplies for their pets or themselves before and after the prompted response section. Before the prompted responses, 25 clients (49.0%) gave between one and four responses. Ten of those clients (40.0%) said they needed grooming services, six clients (24.0%) said they wanted donated supplies to be more accessible, and three clients (12.0%) said they wanted more ER and urgent care options. Other responses included: expanded in-house medication options, expanded dentistry options, delivery/transportation aid, increased human health options, and more streamlined clinic options. After responding to prompted questions, 28 clients (55.0%) gave between one and four open-ended responses. Of those responses, a few items and services repeated from the prompts, however; new responses included four clients (14.3%) who wanted help accessing and managing their pet records, three (10.7%) who wanted help accessing ER and specialist care and three (10.7%) who wanted more help with transporting themselves and pet supplies.

### Overall themes

Many of the respondents said that they wanted pet grooming services at WisCARES. Data for both prompted responses, for which participants needed only to say ‘yes’ or ‘no’, and open-ended responses, for which participants came up with the specific need, are reported separately. Eighty percent of clients (*n* = 41) mentioned grooming either in an open-ended response or in a prompted response. Twenty-seven percent of clients (*n* = 14) replied in an open-ended answer that they would like grooming services of some sort. Many clients used the term ‘groom’ to describe their need, but others offered more exact phrases regarding their need for assistance. These included nail trims, bathing, anal sac expression, and help removing hair mats. One client, who already had a groomer, indicated wanting help keeping their pet from biting the groomer.

Many clients (72.5%) indicated needing help with improving their pet’s dental health through open-ended response and prompted responses. All open-ended responses (9.8% of participants, *n* = 5) used the word “dental” to ask for more services of this nature.

Forty-five percent (*n* = 23) of clients reported that they needed help with transportation either in prompted responses or by expressing difficulty using the bus or securing bus cards, difficulty affording gas, worries around unreliable vehicles, worries about relying on others to give rides, difficulty obtaining supplies from the clinic and difficulty getting animals into the vehicle.

## Discussion

This needs assessment was undertaken to evaluate and improve the services WisCARES offers animals and their humans. Overall, many of the needs that owners expressed in this survey aligned with what WisCARES clinic personnel hear daily while working with clients, and it was useful to see the extent for which services were requested.

### Animal services: pet grooming

For instance, clinic staff were aware that many clients wanted cost effective pet grooming services but would not have predicted that resource to be identified by 80% of survey participants. Although pet grooming may seem to be a luxury, pets without this resource may develop severe medical conditions. McDonald et al. (28) found that across three accessible veterinary service programs, 4–6% of all veterinary medical cases were related to grooming issues and 13% of the ASPCA-NYPD Partnership’s cruelty cases were due to hair matting and related wounds. The American Pet Products Association (29) found that 80% of pet owners groom their pets in an average year. While many people can do this themselves, 30% of respondents in the APPA publication take their dogs to salons, 9% use mobile grooming services, and 8% use retailers, all at considerable expense. In a survey of low-socioeconomic status (SES) pet owners, McDonald et al. (30) found that while 89% of clients reported that regular grooming was important, 92% of owners identified barriers to accessing this service. Expense, along with lack of confidence in grooming their animal themselves, were commonly cited concerns. WisCARES provides basic grooming services regularly. While light grooming may be performed without being charged, in 2024 charges were captured for 535 nail trims, 59 anal sac expressions, and 69 ear cleaning procedures. Twenty-five animals had medically necessary body clipping because of extensive hair mats. Despite the high frequency of these services, increased pet grooming services were identified as a top need of WisCARES clientele indicating a need to expand pet grooming services and increase client awareness of current services and supplies available. Developing a full pet grooming service requires a significant increase in staffing, bathing accessories, boarding and clinic space. Working to improve owner training and confidence in grooming their pet may help decrease the demand for these services.

### Pet supplies: food and treats

The highest prompted pet supply need for dog and cat owners was increased access to food and treats. While the current clinic staff struggle to provide the level of grooming services that clients need for their pets, the clinic has a robust pet food pantry program, thanks to pet food industry partnerships who provide WisCARES with maintenance and medical diets. Through this program, roughly 10 tons of food are moved into the community annually. Many low SES pet owners experience pet food insecurity while also experiencing their own food insecurity. Therefore, owners cope in similar ways by utilizing food pantries, consuming low cost-low value foods or feeding less per meal. Many owners share that they feed their pets before they feed themselves or consider surrendering pets that they cannot feed (31). WisCARES clients also reveal that they offset the costs of pet food or veterinary medical care by using social services like food pantries for themselves. Further understanding is needed to understand why, in the face of ample community resources, pet food remains a highly ranked need. Perhaps clients are unaware of the clinic’s ability to provide affordable food, or they worry that this valuable resource could disappear.

### Animal services: pet dental health options

Seventy-five percent of respondents asked for more dentistry and dental health options for their pets. Many of these responses were indicating a need for preventative dental supplies, however, 10% of respondents requested better access to direct dental care via anesthetized dental procedures. While the current survey did not determine whether respondents know that preventative oral care and anesthetized dental care is offered at WisCARES, it is relevant to note that most owners recognized the need for and wanted more dental care options. While many owners regard dental health as being important in their pets (32), many do not prioritize appropriate dental health strategies (33). Anesthetized dental procedures on pets are often cost prohibitive to WisCARES low-income clientele. Unfortunately, by the time a dental procedure is prioritized, oral disease is often quite severe and extensive dental surgery is warranted. When financial barriers are overcome, other factors such as patient comorbidities and client accessibility prevent another subset of clients from obtaining the procedure. For instance, transportation difficulties and client health concerns can hinder arrival on the pre-determined scheduled procedure day. Based on the interest in prevention expressed in this survey, WisCARES personnel should prioritize discussions of appropriate preventative oral health care, educate owners about dental options and send more dental supplies home with clients to strategize how best to maintain oral health for dogs and cats of all ages.

### Animal services: pet transportation

WisCARES clients struggle with transporting their pets to and from the clinic. This need is often reported to clinic reception staff and 45% of survey respondents requested assistance with transportation. Lack of transportation is a commonly cited difficulty for clients at low-income community veterinary clinics (22, 34). Lack of reliable transportation as a persistent problem for WisCARES clients manifests in different ways. Owning and insuring a car is beyond the means of many clients, and they rely on others to give them rides to where they need to go. Others meet their needs using public transit or transportation services specifically designed for human medical appointments or for senior citizens. Animals are typically not permitted by these services, making it difficult for people without cars to bring their companion animals to a veterinary appointment. Increasing access to mobile veterinary practices and telemedicine appointment options, in addition to encouraging policy changes to increase allowance of pets on public transportation have been suggested as a possible solution to this problem (22, 33, 35).

### Human social services

A majority of respondents were interested in learning about local agencies and social services. These organizations are able to provide tangible items, like clothing and gear for winter and summer months, personal hygiene supplies, and food. They also provide services, such as locating and navigating health and mental health care, legal assistance, job search resources, and childcare.

WisCARES clients often have challenges finding stable shelter and permanent housing. Being admitted to a shelter is one of the first tasks for many people when they lose their housing. This is difficult under the best of circumstances, and even more so with an animal family member. Most sleeping and day-use shelters in the U.S. prohibit animals, forcing people experiencing homelessness to choose between sleeping indoors and maintaining their relationship with their companion animal (36, 37). Survey responses asking for foster care and boarding services reflect this dilemma.

Many WisCARES clients live in rental housing. They have managed to overcome myriad barriers, such as low income, an expensive rental market, lengthy waits for subsidized housing, a record of prior evictions, and property owners fearing that animals will damage their building or harm other residents (38, 39). Respondents requested help talking with landlords about these issues, as well as behavioral consultation and training classes to teach their animals to live successfully in these settings.

Low-income and unhoused people may struggle to find sufficient nutritious food because of their lack of financial resources, not qualifying for nutrition assistance, or living in food deserts. Nationally, an estimated 57% of homeless and housing insecure people also experience food insecurity (40), meaning that they do not know where they can reliably access food for themselves or their families. Forty-one percent of survey respondents said that they wanted to know more about food support options in the community, such as food pantries, free meal programs, and signing up for federal and state nutrition programs. It is well documented that low-income and unhoused pet owners tend to provide food for their animals first, leaving less money to buy their own food (41).

As a social determinant of health, social support is recognized as being important to both physical and mental wellbeing (42). Unhoused people experience higher levels of social isolation and lower levels of social support than the general population (43, 44), placing them at risk for poorer outcomes in a variety of areas, including health, housing disruption, and the likelihood of being assaulted. Over WisCARES’ 11 years of delivering care, we have become accustomed to some clients bringing their animals in for wellness appointments where no vaccines, food or products are needed or to address concerns which end up being minor or even “normal” findings for their pet. Although they do not receive treatment at these visits, clients have conversations with clinic staff who know them by name, ask after their welfare, and express care. The human health care literature has explored patients’ use of health care appointments to engage in social interaction and decrease loneliness (45, 46), finding it a relatively common occurrence. Gerst-Emerson & Jayamardhana cite the Campaign to End Loneliness’ finding (47) that one in ten medical visits to family physicians in the UK may be for social rather than medical reasons. In our survey, nearly 40% of survey respondents said that they would be interested in spending time with other WisCARES clients in a discussion forum. Relationships and social support are clearly important to WisCARES clients, and we are investigating interventions to meet this need.

### Obtaining supplies

Many WisCARES clients reported obtaining pet supplies from large commercial chain stores (53% of responses) such as generalized retailers (Walmart, Family Dollar, etc.) or pet supply retailers (PetSmart, Petco, etc.). Twenty-six percent of respondents purchase or receive supplies for their pets from WisCARES, other local support agencies (The Humane Society, Pets for Life, etc.) or from friends and family. Utilization of large commercial retailers and aid agencies is consistent with known trends among low-SES groups (48, 49). Only 13% of respondents to the open-ended questions indicated that they purchase food from online retailers. In a review of online supply purchasing trends, Ghosh (50) theorizes that people with lower incomes are discouraged from purchasing goods online due to low levels of trust, low tolerance for risk and a perception that purchasing online is more complicated. Once these factors are accounted for, many income disparities in the use of online platforms disappear. At WisCARES, this information may be useful to consider when staff recommend that our clients order supplies or medicine from online retailers. Compliance may be low and there may be more barriers to success than have been previously considered.

### Records consistency

Many clients, especially those in low-SES households, do not seek veterinary care regularly for all of their pets (51) and those that do, seek it more frequently for dogs than for cats (52). The number of pets in a household was evaluated to determine if the subset of WisCARES clients who took this survey were bringing all their pets to the clinic or if there were record inconsistencies, and pets at home were hidden from the medical team. This assessment provided an opportunity to see if the clinic was missing large numbers of animals that may need wellness assessments or medical contact points. Fortunately, the variation was small for the clients that were surveyed, however if 7.8% of clients who have a variation of two or more pets is representative of the larger WisCARES client population, it may be helpful to routinely ask clients at intake how many pets they currently have and whether they need services for those animals as well.

One unexpected finding of this study was the high rate of new phone numbers for people who had not visited the clinic for more than a year. In hindsight, WisCARES clinic staff recognized that a barrier to providing consistent care is not having updated client phone numbers. A study on phone use in women experiencing homelessness showed that 50% of study participants had multiple phones in a 1-year period (53) and general trends around cell phone use in lower-SES groups show that while smart-phones are immensely helpful, patterns of inequity are evident around phone consistency and connectivity (54). Hence, dedication to confirming contact information at every visit is crucial. It may be beneficial to routinely take notes about the best ways to contact individuals and to think creatively regarding the use of secure communication apps.

This study is limited in that it looks at the needs of a small number of clients in a community where WisCARES has been in operation for over 10 years. If this survey had been given to pet owners in a place where access to resources was much more limited, the answers may reflect different needs. All community medicine practitioners must start with knowing one’s community as the needs of one community may look different from the needs of another community. The needs of WisCARES clients in Madison, Wisconsin may not reflect those of another city in the United States. Lastly, many open-ended responses voiced concerns that were only held by one or two people and were not included in the current document. While these have help shaped conversations about internal protocol development of WisCARES, they are too niche to report here.

Overall, this assessment has confirmed many of the beliefs that WisCARES personnel had about resources that clients need for their pets. The client survey responses validate these beliefs, showing that WisCARES clients are thinking about certain services more than was recognized by clinic staff. Other responses have added clarification and nuance, improving the understanding of what is of interest to WisCARES clients. Analyzing all the client responses in this way has helped WisCARES staff understand more about client awareness (or lack of awareness) of services that are already offered. Intentional and structured data gathering has helped staff understand more specific client and patient needs, which are likely to reflect the needs of the larger community of clients who come to WisCARES. In summary, this assessment has provided the staff at WisCARES Community Clinic with measurable goals and reference points for further development and expansion of our Community Resource Center to better serve the local community of companion animal caretakers.

## Data Availability

The raw data supporting the conclusions of this article will be made available by the authors, without undue reservation.
