# Supplementary material for: Assessing client needs in community veterinary care: a case study from WisCARES
Source: Front Vet Sci. 2025 Oct 13;12:1675984. doi: 10.3389/fvets.2025.1675984 (PMC12557790; doi:10.3389/fvets.2025.1675984)
Supplement: Supplementary file 1 [file Table_1.docx]

**Supplementary Appendix** **– Survey Questions**

**Question 1:** How many pets do you own? 
*If the respondent needs clarity, ask, "How many animals live with you that you are financially responsible for?" or "How many animals live with you and would move to another residence with you if you moved?"*

*Surveyor was asked to collect number of pets of each species type, if that information was not volunteered in the initial response.*

**Question 2:** People get the things they need for their pets from all kinds of places. In the past year, what kinds of stores, clinics, agencies, family, or friends have you gone to, to get the things you need for your pet?

*Surveyor was asked to write down each unique place that was mentioned.*

**Question 3:** If WisCARES were to expand the services we offer, what do you think should be included?

*Surveyor was asked to write each recommended service with necessary elaboration, but not to document comments that were not related to services.*

**Question 4a:** *Skip if no cats in the household:*

Which of these **CAT** supplies would you personally use if they were available at WisCARES?

- Cat food pantry
- Treats
- Food/water bowls
- Cat litter/cat boxes
- Collar/harnesses
- Toys
- Bed
- Carrier/crates
- Scratching pad/board/posts
- Preventative dental supplies
- Nail trimmers
- Cat trees

**Cat supplies:**
Is there anything that we forgot?  

*Record any additional thoughts or elaborations that the client provided from the pre-listed items.*

**Question 4b**. *Skip if no dogs in the household:*

Which of these **DOG** supplies would you personally use if they were available at WisCARES?

- Dog food pantry
- Treats
- Food/water bowls
- Leash
- Outdoor apparel
- Toys
- Bed
- Carrier/crate
- Preventative dental supplies
- Basic shampoo or cleaners
- Nail trimmers
- Hair clippers
- Pet diapers

**Dog supplies:**
Is there anything that we forgot?  

*Record any additional thoughts or elaborations that the client provided from the pre-listed items.*

**Question 4c:** Which of these **animal services**would you personally use if they were available at WisCARES? 

*If yes to any services, ask for specifics:*

In what ways do you envision [type of service] benefiting you and/or your pet?

- Dog training classes (classes where you learn how to train your dogs to obey basic commands)
- Consultation with an animal behaviorist (sessions with a behaviorist to learn how to treat issues like fear, anxiety or aggression in your pet)
- Pet grooming (nail trims, bathing, clipping, ear cleaning)
- Temporary foster care (have the ability to place your pet with another family for a few weeks/months during difficult times)
- Boarding (have the ability to keep your pet at WisCARES for a few days during difficult times)
- Doggy day care services (have the ability to keep your dog under supervision and enriched during a work day)

**Animal services:**
Is there anything that we forgot?

*Record any additional thoughts or elaborations that the client provided from the pre-listed items.*

**Question 4d:** Which of these **human services and supplies** would you personally use if they were available at WisCARES?

*If yes, ask what type of service within this category would help and how would it help them*

- Finding out about local agencies and social services that can help with problems you're facing
- Opportunity to join a discussion forum with other WisCARES clients
- Help talking with landlords
- Navigate medical appointments, physical therapy, or mental health resources
- Food support
- Finding child daycare support
- Help with navigating job interviews
- Legal resources
- Transportation support
- Personal hygiene supplies and toiletries
- Cold weather supplies like thermal socks, long underwear and hand warmers
- Warm weather supplies like sunscreen, water bottles and lip balm

**Human services and supplies:**

Is there anything that we forgot?

*Record any additional thoughts or elaborations that the client provided from the pre-listed items.*

**Question 5:** In addition to what we’ve just talked about, what services did you or your pet need this year that you wish you had better access to?

*Surveyor was asked to write each recommended service with necessary elaboration, but not to document comments that were not related to services.*
